# A review of circular RNAs in colorectal cancer: insights into biomarker discovery and therapeutic targeting

**DOI:** 10.3389/fonc.2025.1549046

**Published:** 2025-07-04

**Authors:** Sibin Nambidi, Karunakaran Bhuvaneswari Shruthi, Sujatha Padmanaban Kanimozhe, Antara Banerjee, Suresh Babu Kondaveeti, Asim K. Duttaroy, Surajit Pathak

**Affiliations:** ^1^ Medical Biotechnology Lab, Faculty of Allied Health Sciences, Chettinad Academy of Research and Education (CARE), Chettinad Hospital and Research Institute (CHRI), Chennai, India; ^2^ Department of Biochemistry, Symbiosis Medical College For Women, Symbiosis International (Deemed University), Pune, India; ^3^ Department of Nutrition, Institute of Basic Medical Sciences, Faculty of Medicine, University of Oslo, Oslo, Norway

**Keywords:** colorectal cancer, circular RNA, noncoding RNA, diagnosis, biomarker, prognosis, miRNA sponges

## Abstract

Colorectal cancer (CRC) is one of the most common cancers, with an incidence that has increased significantly over the last 20 years. The World Health Organization (WHO), under its cancer program, identifies CRC as the third most prevalent cancer worldwide with a high mortality rate, especially in patients under the age of 50. Despite advances in surgery, chemotherapy, radiotherapy, and molecular targeted therapy, CRC shares a low survival rate and poor prognosis due to late diagnosis. To address these challenges, research into alternative diagnostic and therapeutic strategies has increasingly focused on molecular mechanisms, including noncoding RNAs (ncRNAs). Circular RNAs (circRNAs), a subclass of endogenous ncRNAs characterized by their covalently enclosed loop structure, demonstrate greater stability than linear RNAs, making them potential candidates for clinical applications. The circRNAs possess differential expressions in cancers and function as tumor suppressive or oncogenic activities. This review discusses the recent findings on circRNAs and their potential for biomarkers and therapeutic targets in CRC. As circRNAs can serve as miRNA sponges, increase or decrease mRNA expression, and function to regulate an oncogenic or tumor suppressor pathway, there may be the possibility for an early diagnosis, prognosis, or therapeutic role of circRNAs in CRC. Highlighting the clinical implications of circRNAs, this review discusses their potential to transform current CRC management strategies and address critical gaps in timely diagnosis and effective treatment. It also emphasized the need for further clinical research to validate their utility and translate these findings into practice.

## Graphical Abstract

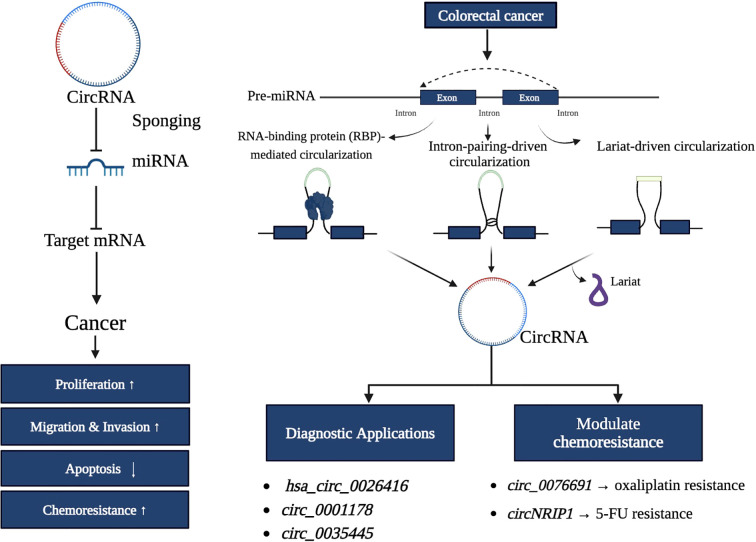

## 1 Overview of the CRC

Colorectal cancer (CRC) is the third most commonly diagnosed cancer and continues to be a leading cause of cancer-related deaths (1). In 2020, there were approximately 1.93 million new cases of CRC reported globally, resulting in nearly 0.94 million deaths. The projected number of new CRC cases worldwide will be 3.2 million by 2040 (2). The development of CRC is influenced by various interactions between genetic and environmental factors, including nutrition, diet, physical inactivity, insulin-like growth factors, and supplements (3). About 20% of colon or rectal tumors are believed to emerge from an inherited mutation in genetic code, including familial adenomatous polyposis (FAP), hereditary non-polyposis 5colorectal cancer (HNPCC), and tumors with a significant family history (4, 5). Obesity has also been shown to be a considerable risk factor in CRC development, and studies repeatedly show that physically inactive individuals are possibly prone to CRC (6). A diet high in protein, red meat, and alcohol and low in fruits, vegetables, and dairy foods further enhances CRC risk. Johnson C.M., et al. (2013) performed a meta-analysis. He summarized a number of modifiable risk factors for CRC incidence, which included higher BMI, cigarette smoking, red meat intake, low fruit and vegetable intake, post-menopausal hormone therapy, and inflammatory bowel disease (IBD) (7).

Early diagnosis is crucial for improving patient outcomes and overall survival rates in CRC (8). As such, discovering new biomarkers and developing better treatment strategies are essential because they will allow for early detection, personalized treatment, and ultimately better management of CRC for better prognoses and quality of life (8). The Centers for Disease Control and Prevention estimates that in 2024, there will be about 97,220 new colon cancer cases and about 43,030 new rectal cancer cases diagnosed in the United States (9). However, the incidence of CRC has been declining for the last few decades. The incidence rate in the United States went from 60.5 cases per 100,000 in 1976 to 46.4 in 2005, and 40.7 between 2009 and 2013 (9). Although this overall trend does not hold for younger populations, between 2003 and 2012, the incidence of CRC declined by approximately 3% annually, and the overall mortality decreased by 51% from its peak in 1976 until 2014. Overall, this progress can mainly be attributed to increased detection via screening and improved therapeutic interventions (9).

It is predicted that in the United States, the incidence rates for colon and rectal cancer for people ages 20–34 years could increase by 90% and 124%, respectively, by the year 2030. For people ages 35–49 years, incidence rates could increase by 27.7% and 46%, respectively (10, 11). CRC is most prevalent in industrialized countries, but incidence rates are rising in low and middle-income countries due to the growing propensity to adopt Western dietary and lifestyle measures (12). The highest incidence rates by country are Australia and New Zealand, followed by European countries and North America (12). Central Eastern Europe demonstrates some of the highest mortality rates for any cancer. Conversely, South Asia and Africa display the lowest incidence and mortality rates but face higher mortality-to-incidence ratios due to a lack of access to screenings and treatments (12). In Italy, CRC incidence increased among men from 30 to 70 per 100,000 between 1970 and 2010, while among women, incidence rates stabilized at approximately 38 per 100,000 in the late 1990s (13). Colon cancer (CC) is more common than rectal cancer (RC) in developed countries, though markedly more common among women (14). Thus, CC is more prevalent than RC (ratios as high as 2 or 3:1). In Europe, there are more than 250,000 new cases of CC each year, corresponding to around 9% of cases diagnosed yearly. Urbanization and industrialization have been key contributors to the increasingly high incidence of CRC cases in developed countries (14, 15). Globally, CRC incidence remains low in Africa and Asia. North America, Europe, and Australia are considered high-risk regions, while Central and South America, Asia, and Africa are classified as low-risk areas (16, 17). CRC prevalence significantly increases with age, rising from approximately 20 cases per 100,000 per year in individuals aged 45–54 years to 55 per 100,000 in those aged 55–64 years, 150 per 100,000 in the 65–74 age group, and 250 per 100,000 among individuals over 75 years (18). CRC survival rates have improved over time, especially in Europe, but with important regional discrepancies; for example, by the late 1990s, the five-year survival rate rose to 54%, although the rate was notably lower in Europe than the higher reported rates in the US (19, 20). The incidence of CRC in Italy in the North was double compared with the South in men and women (21, 22).

Adenocarcinoma is the predominant histological type of colon cancer and makes up 92–95% of tumors in the bowel. Mucinous or colloid adenocarcinomas make up about 17% of tumors in the bowel (23). The rare epithelial cancers (adeno-squamous carcinomas and squamous cell carcinomas (adenoacanthoma) are rarely seen. CRC lesions are assessed for differentiation (well, moderate, poorly), and differentiation is assessed via standard glandular morphology and cytology (23). Poorly differentiated cancers tend to carry a worse prognosis, often secondary to genetic defects, but it is unclear what mutations correlate with this differentiation. Recognizable differentiation was absent in 20–25% of colon malignancies. Polyps, commonly designated precursor lesions for CRC, are mainly recognized in the lower GI tract, the rectum or appendix, but can happen anywhere in the colon (24). Recent developments in the molecular pathophysiology of CRC have led to the opportunity to identify several aspects that can be considered as potential biomarkers, genetic as well as epigenetic and protein-based markers from fecal, serum, and tissue samples. Some examples of key research areas include DNA methylation, microRNAs, circular RNAs, long non-coding RNAs, and exosomes (25).

Although various imaging techniques are available, CT imaging remains the most widely accessible, reliable, and versatile tool for cancer staging and assessing local tumor characteristics (26). While PET/CT colonography, a combination of PET and CT imaging, has also shown great potential for cancer detection and staging. Its proficiency in identifying metastatic lymph nodes is of particular importance in rectal cancer management, in which it is more common for patients to undergo preoperative treatment with node-positive disease (27). Sigmoidoscopy and colonoscopy are utilized extensively for both screening and diagnostic purposes (28). The current most utilized diagnostic biomarker for CRC is the fecal immunochemical test (FIT), the foundation of population-based CRC screening programs intended to reduce CRC mortality (29). Fecal immunochemical tests are also used to diagnose in symptomatic patients and to perform surveillance following adenoma removal (30). FIT’s ability to detect human hemoglobin in stool using antibodies and without necessitating dietary restrictions results in greater participation in screening programs (31). The guaiac fecal occult blood test (g-FOBT) is another commonly utilized method for detecting blood in stool and is a chemical test that relies on peroxidase activity. g-FOBT tests are inexpensive, relatively easy to perform, and readily available (32). Many randomized controlled studies have shown that g-FOBT tests can decrease CRC mortality rates due to tumors being identified earlier. However, there is concern that g-FOBT tests with limited sensitivity for detecting colorectal adenomas may fail to prevent the onset of CRC (33).

Choosing an appropriate therapeutic strategy and determining the dosing regimen continue to be a large challenge in cancer therapy. For CRC, there are different modes of treatment, including surgical intervention, cryosurgery, chemotherapy, radiation therapy, and targeted therapies (34). Physicians use chemotherapy most frequently, which is the administration of different drugs that block cancer cell growth. Still, often, these agents have adverse effects like chemotherapy-induced leukopenia, fatigue, palmar-plantar erythrodysesthesia, chemotherapy-induced gastrointestinal toxicity, chemotherapy-induced oral mucositis, nausea, vomiting, aches, anemia, hematologic complications, and liver impairment (35). Side effects diminish the quality of life, lessen the efficacy of treatment, and cause drug resistance over time. Surgical intervention remains the most essential treatment for CRC, addressing approximately 50% of cases successfully, where recurrence of the cancer after surgery remains one major limitation, leading to poor outcomes and often causing death (34, 35). Even with early detection and treatment advances, patient clinical outcomes remain unfavorable. While radiation and chemotherapy are standard approaches, these methods of treatment have limitations, reinforcing the need for more efficient alternative therapies. As a result, there are more efforts to establish molecular biomarkers for diagnostic and therapeutic purposes (35). There is an urgent need to develop better diagnostic and therapeutic approaches while understanding the molecular mechanisms of CRC progression (36).

## 2 Role of CircRNAs

The first single-stranded, covalently closed circRNAs were identified in human HeLa cells using electron microscopy in 1979 (37). Subsequent research revealed that numerous species, including viruses, prokaryotic cells, single-celled eukaryotes, and mammals, have evolved to produce circular forms of RNA. High-throughput RNA sequencing and bioinformatics analyses have established circRNAs as a ubiquitous component of the human transcriptome, present across many metazoans (38). CircRNAs exhibit greater stability than linear RNAs due to their resistance to RNases (39). This inherent stability and distinct expression patterns have established circRNAs as biomarkers and potential therapeutic targets, particularly in cancer (39). CircRNAs perform diverse cellular functions, including serving as protein scaffolds, miRNA sponges, and templates for polypeptide translation (40). Despite their biological significance, the precise mechanisms underlying their aberrant expression in diseases such as cancer remain incompletely understood. CircRNAs regulate physiological and pathological processes through interactions with cellular components, often indirectly modulating gene expression. For instance, circRNAs can act as competitive endogenous RNAs (ceRNAs) by sequestering miRNAs, freeing mRNA targets, and influencing critical signaling pathways (40).

**Figure 1:**
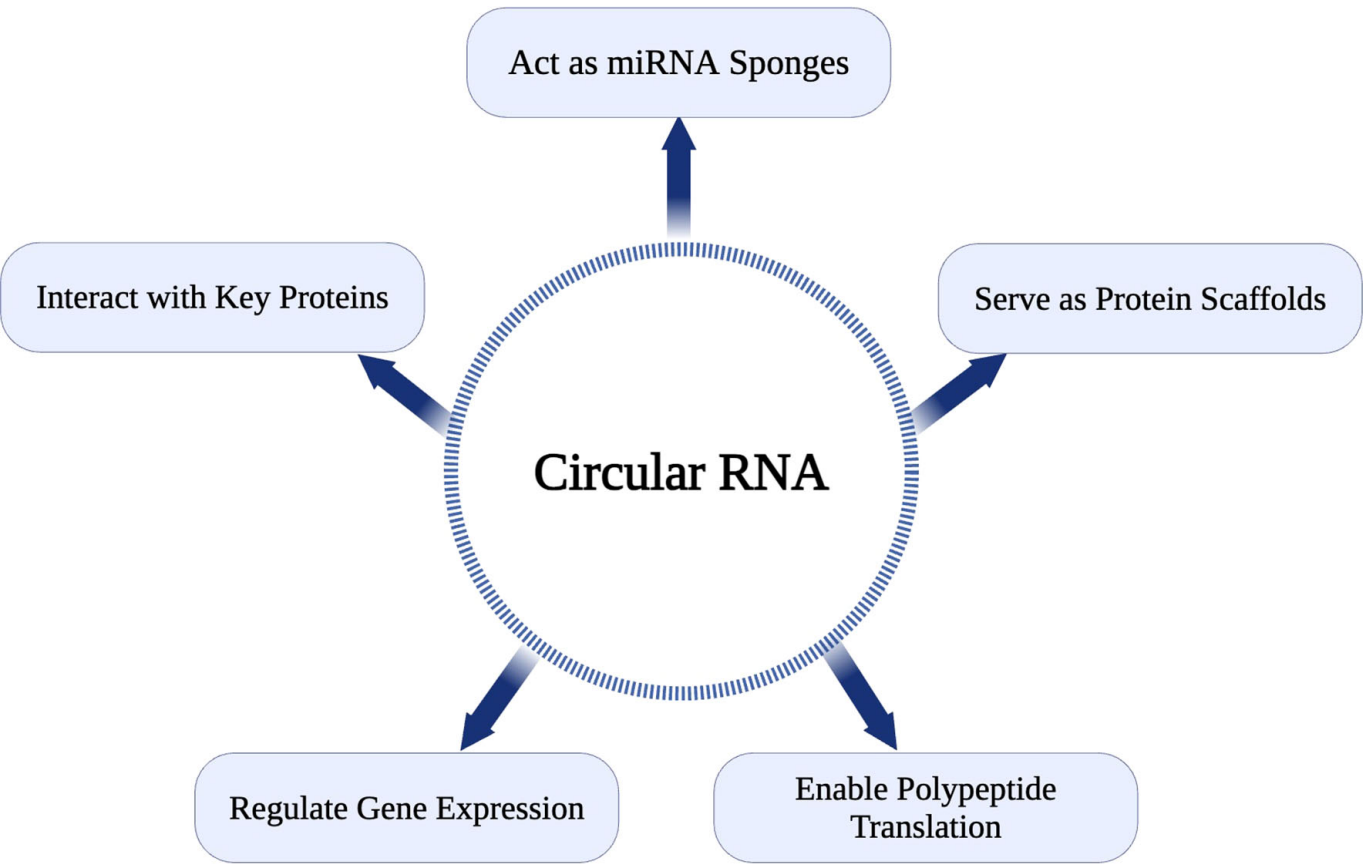
Functional roles of circRNAs in cellular processes.

Detection and quantification of circRNAs involve northern blotting, RT-qPCR with divergent primers, RNA sequencing (RNA-seq), circRNA profiling, and *in situ* hybridization targeting the back-splice junction (41). While advances in RNA sequencing technologies have enhanced circRNA identification, challenges remain, such as distinguishing circular from linear transcripts and elucidating their biological roles (42). CDR1as, the antisense transcript of CDR1, a circRNA, was among the first circRNAs investigated for its functional significance. Recent findings indicate that CDR1 interacts with p53 to inhibit pro-metastatic activities by preventing its binding to MDM2 (42). This interaction highlights the ability of circRNAs to influence key regulatory proteins, underscoring their profound potential in tumor biology. The diverse function of CircRNA is represented in **Figure 1**.

### 2.1 Classification and types of circRNAs

Depending on their genomic origin, circRNAs are organized into various categories: exonic circ RNAs (ecircRNAs), intronic circRNAs (ciRNAs), exon-intron circRNAs (EI ciRNAs), intergenic circRNAs, and tRNA-derived circRNAs (triRNAs) (43). Exonic circRNAs are derived from the exonic sequences of protein-coding genes. They are primarily cytoplasmic, often acting as miRNA sponges to impact gene expression in the post-transcriptional phase (44). Intronic circRNAs are formed at in the intronic region and are located in the nucleus, with ciRNAs being reported to regulate transcription. Exon-intron circRNAs are formed from the components of both exons and introns and can exist in both nuclear and cytoplasmic compartments. The functionality of exon-intron circRNAs is variable in their precise composition and cellular location (43, 44). Intergenic circRNAs originate from non-coding genome regions between annotated genes and can act as a further regulatory layer. Furthermore, a subset of circRNAs is thought to come from tRNA sequences called triRNAs (44). These circRNA subtypes involve different cellular biology processes, including gene regulation and protein interactions. They are potentially relevant to the pathology of many human diseases, indicating their biological and clinical significance (44, 45).

The ecircRNAs, intronic circRNAs, and EIciRNAs are diverse in their cellular localization and functions (45). EcircRNAs are often cytoplasmic and serve primarily as miRNA sponges, sequestering these regulatory miRNAs from influencing their mRNA targets (45). On the other hand, ciRNAs and EIciRNAs are predominatly located within the nucleus and regulate their parental genes transcriptionally. CirRNAs can interact with RNA polymerase II and transcription factors to regulate expression and EIciRNAs not only regulate transcription of their source genes but also interact with RNA polymerase II and the U1 snRNP complex, indicating a role in transcriptional activation (46, 47).

### 2.2 Biogenesis of circRNAs

Since circRNAs are formed from pre-mRNAs, the standard spliceosome machinery may also regulate their synthesis. The biogenesis of circRNAs and their mechanisms of action remain incompletely understood. While exon skipping is considered a key regulator of circRNA production through alternative splicing, the exact primary process driving circRNA generation has yet to be determined (48). Three distinct theories have been proposed to explain the synthesis of exonic circRNAs, which are the dominant type in eukaryotes: Lariat-driven circularization (exon skipping), Intron-pairing-driven circularization (back splicing), and RNA-binding protein (RBP)-mediated circularization (24).

**Figure 2:**
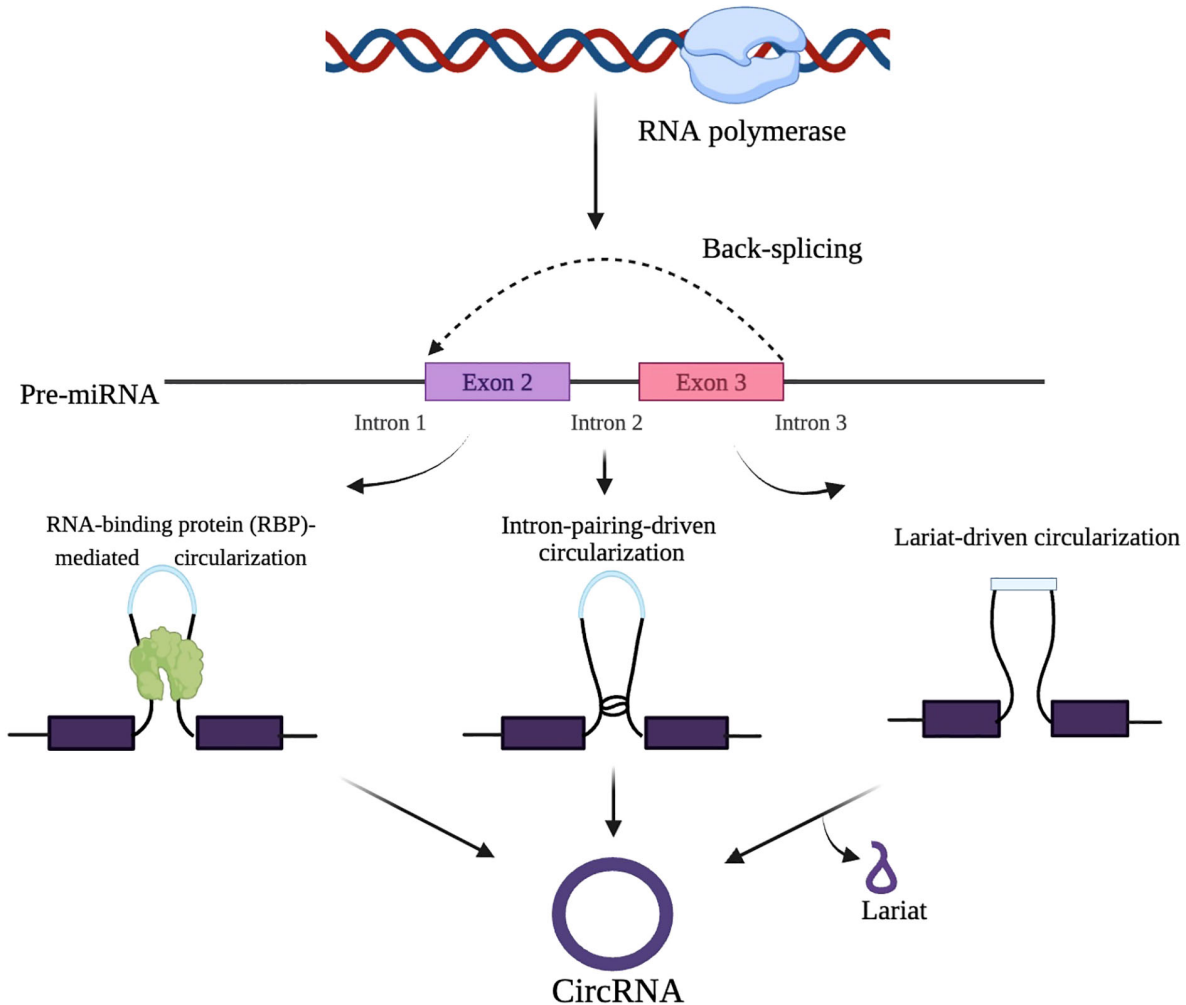
Mechanisms of circRNA biogenesis. CircRNAs are formed through the back-splicing of precursor mRNA via RNA-binding proteins, intron pairing, or lariat-driven circularization.

In exon skipping, non-canonical ‘back splicing’ processes are thought to occur during RNA synthesis. For instance, an early study on the human cytochrome P450 2C18 gene reported a connection between exon skipping and circular RNA isoforms, identifying four alternative circularization patterns and their associated exon-skipped transcripts (49). Back splicing involves a bridging mechanism that reduces the distance between the splice sites of neighboring introns, facilitating the formation of circRNAs. Additionally, the splicing process utilizes specific elements, such as an 11-nucleotide GU-rich sequence at the 5′ splice site and a 7-nucleotide C-rich element at the 3′ branch point of introns, which promote circRNA synthesis (34, 49). A study by Li et al. (2024) identified ZC3H14, a conserved RNA-binding protein, as a new regulator of circRNA biogenesis. ZC3H14 promotes back-splicing binding at exon-intron boundaries (EIBs) and 3’ untranslated regions (UTRs) to encourage recruitment of the spliceosome by dimerization (50). Furthermore, ZC3H14 does not directly impact BSS–3’UTR distance, suggesting that ZC3H14 may play a prominent post-transcriptional silencing role in regulating circRNA biogenesis. The strong binding of ZC3H14 at 3’UTRs stabilizes the interaction with EIBs, bringing splice sites closer and increasing the likelihood of circularization (50). Therefore, these findings expand the current knowledge about the regulation of circRNA biogenesis beyond the previously defined intronic repeat element (50). **Figure 2** illustrates the process of pre-mRNA to circRNA, incorporating the spliceosome machinery and three primary circularization mechanisms: RNA-binding protein-mediated circularization, intron-pairing circularization, and lariat circularization. In the lariat circularization mechanism, exon-skipping gives rise to a lariat that is further processed into circRNA. These mechanisms facilitate back-splicing, resulting in covalently stabilized or closed circRNAs forming.

**Figure 3:**
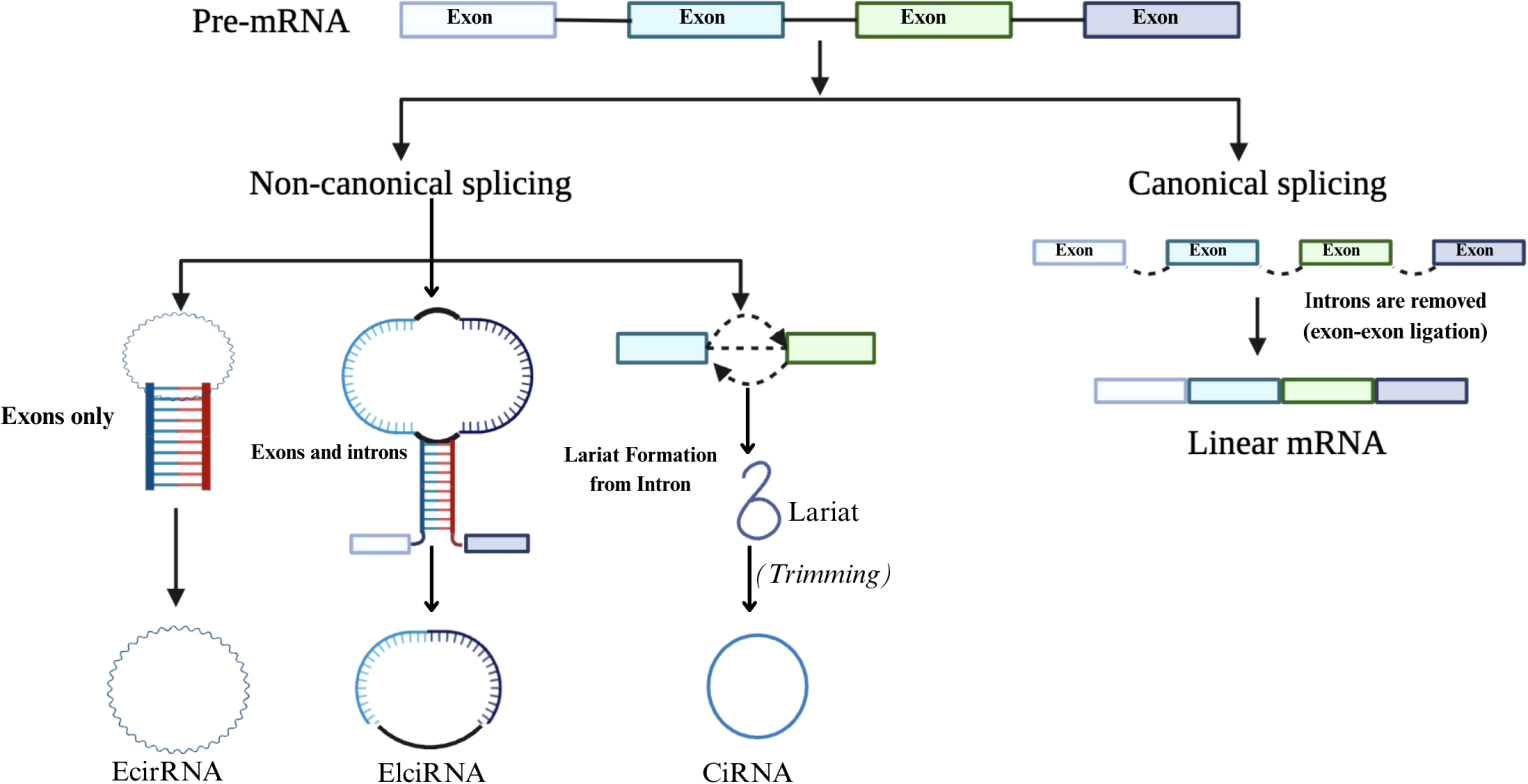
Canonical and non-canonical splicing pathways. Pre-mRNA undergoes canonical splicing to form linear mRNA, or non-canonical splicing to generate circRNAs such as EciRNA, ElciRNA, and CiRNA via different circularization mechanisms.

It was recently discovered that circRNAs are formed through a back-splicing event during pre-mRNA processing. CircRNAs are considered a specialized product of RNA processing, where spliceosome activity preferentially removes introns from the ring region (51). Unlike canonical splicing, which joins exons linearly, back-splicing covalently links a downstream 5′ splice site to an upstream 3′ splice site, forming a stable circular structure (52). CircRNAs play critical roles in CRC, influencing cell growth, invasion, movement, and apoptosis. Depending on the context, they can function as tumor suppressors or oncogenes (53). This dual functionality is shaped by their interactions with miRNAs, RNA-binding proteins, and chromatin regulators. For example, circRNAs bind and sponge oncogenic or tumor-suppressive miRNAs, thereby modulating the expression of downstream target genes involved in key oncogenic pathways. This miRNA sponge activity regulates gene expression and contributes to chemoresistance and metastasis in CRC (54, 55). **Figure 3** illustrates the processing pathways for generating linear mRNA and circRNA from a single pre-mRNA molecule. Canonical splicing produces linear mRNA by joining exons in a sequential order.

## 3 Role of circRNA in CRC diagnosis

**Figure 4:**
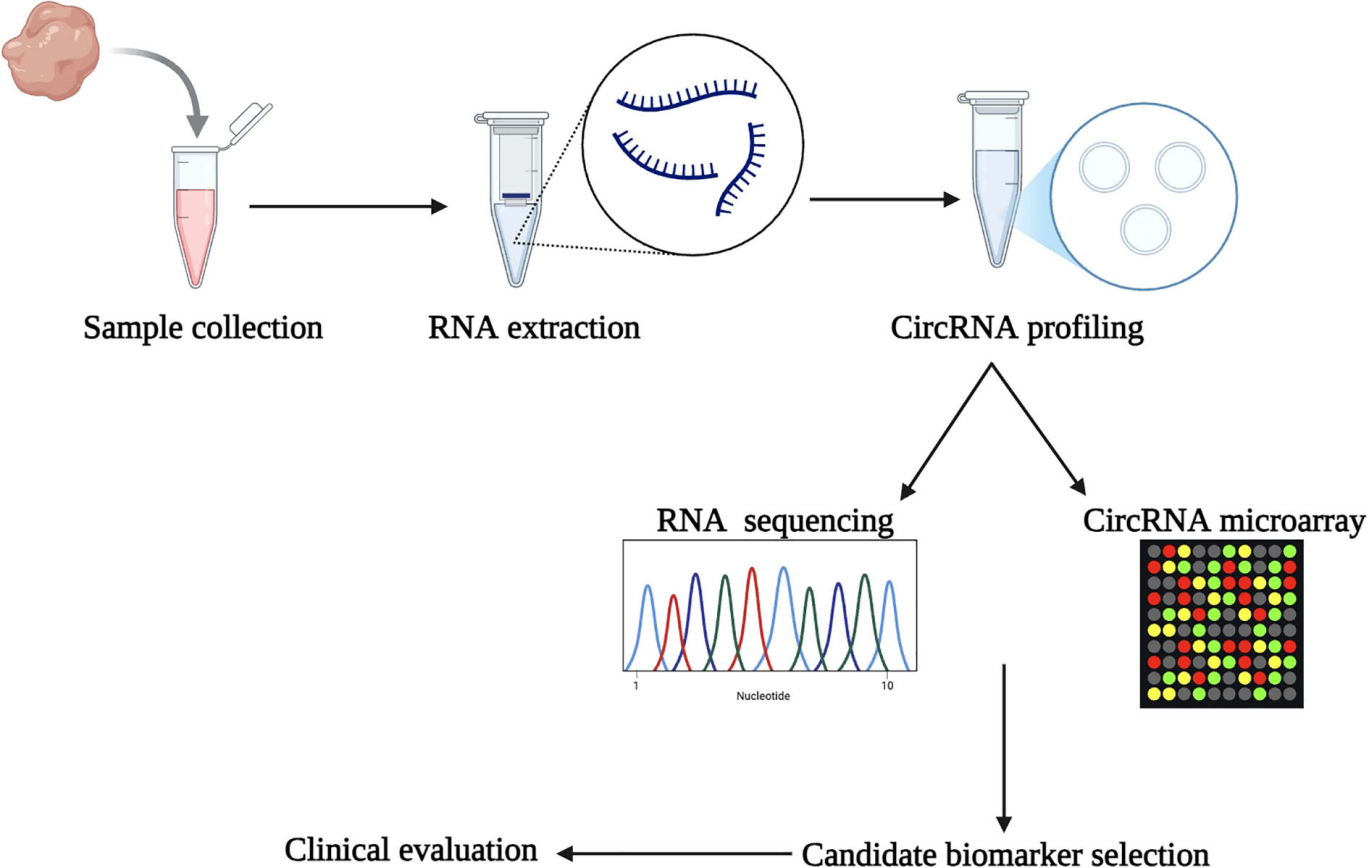
Workflow for circRNA biomarker discovery. The process begins with sample collection and RNA extraction, followed by circRNA profiling using RNA-Seq or circRNA microarrays. Candidate biomarkers are identified and evaluated clinically, leading to the development of diagnostic tools.

CircRNAs are a significant diagnostic biomarker due to their conservative nature, abundance, and tissue-specificity (56). Two RNA types are contained in the circRNA-microRNA code, and they work together to control the expression of genes. As a result, cancer can be predicted early (57). circRNAs have potential use in monitoring therapy effectiveness and determining cancer prognosis (58). Understanding circRNAs and their expression patterns could eventually result in the development of biomarkers for cancer diagnosis, including colon, breast, gastric, lung, and hepatocellular carcinoma (58). **Figure 4** shows the systematic workflow for discovering biomarkers using circRNAs and developing diagnostic tools. The first phase consists of subject sample collection, RNA extraction including circRNAs, and then circRNA profiling using RNA sequencing (RNA-Seq) or circRNA microarrays. Candidate circRNA biomarkers are identified based on their differential expression profiles and clinical relevance for potential use as a diagnostic or prognostic tool. The clinical samples from which circRNA biomarkers were selected will be used to undergo clinical evaluation for their diagnostic or prognostic potential.

CircRNAs play a significant role in the initiation and progression of cancer. The formation of circRNAs is believed to result from the binding of RNA-binding proteins and specific repeating sequences in the introns adjacent to the circularizing exons (59). CircRNAs are thought to influence cancer development through multiple mechanisms, including interacting with proteins, sponging miRNAs, altering gene transcription or splicing, and even translating into proteins or short peptides (60). Abnormal expression patterns of circRNAs have been linked to the development of various human malignancies, including colon cancer. Several circRNAs have been identified as abnormally expressed in CRC tissues, regulating carcinogenesis (61). The circRNA circPPP1R12A is highly expressed in CRC patient tissues, and its overexpression correlates with a lower life expectancy (62). Similarly, CRC tissues show overexpression of circPIP5K1A, which promotes cell proliferation and invasion while reducing the expression of the corresponding protein, thereby impairing cell viability. Consequently, circPIP5K1A enhances colon oncogenesis by blocking miR-1273a. Furthermore, higher circCTIC1 expression in CRC tissues has been associated with poor tumor prognosis. CircRNAs can regulate gene expression at multiple levels, as seen in their potential to be translated into peptides or proteins (63).

circRNAs play a pivotal role in CRC progression by interacting with various proteins and miRNAs, thereby influencing key tumorigenic processes such as proliferation, migration, invasion, and epithelial-mesenchymal transition (63). These molecules serve as critical regulators of gene expression, acting as sponges for specific miRNAs, and participate in modulating signaling pathways associated with cancer progression (64). For instance, circ-0053277, significantly upregulated in CRC tissues, functions as a sponge for miR-2467-3p. By sequestering miR-2467-3p, circ-0053277 enhances cell migration, proliferation, and EMT, contributing to increased metastatic potential and poorer clinical outcomes in CRC patients (65). These findings highlight circ-0053277’s role as an oncogenic factor, underscoring its potential as a therapeutic target. Thus, inhibiting circ-0053277 could offer novel strategies to reduce metastatic spread and improve patient prognosis, especially in advanced CRC (64, 65).

Similarly, hsa_circ_0026416 is another circRNA overexpressed in CRC tissues and plasma, acting as a sponge for miR-346 (66). Functional studies have demonstrated its role in promoting CRC growth in both *in vivo* and *in vitro* models, emphasizing its contribution to tumorigenesis. Importantly, detecting hsa_circ_0026416 in plasma positions it as a promising non-invasive biomarker. Its potential use in liquid biopsies offers opportunities for early diagnosis, real-time disease monitoring, and personalized therapeutic interventions (66). Additionally, circ-000166 exhibits elevated expression levels in human CRC tissues and colon cancer cell lines (67). This circRNA has been implicated in aggressive cancer behaviors, including enhanced cell migration and invasion, making it a potential target for therapeutic intervention (67). Given its association with advanced CRC stages, targeting circ-000166 could be particularly beneficial for managing aggressive and treatment-resistant cases (67). Collectively, circRNAs demonstrate multifaceted roles in CRC pathogenesis, acting as oncogenic regulators and potential diagnostic or therapeutic targets. Their interactions with miRNAs and proteins, coupled with their detectability in tissues and bodily fluids, establish circRNAs as integral components in the molecular aspect of CRC. Future research focusing on the therapeutic targeting and diagnostic utility of circRNAs, such as Circ-0053277, hsa_circ_0026416, and circ-000166, could pave the way for innovative approaches to CRC management (68).

## 4 Therapeutic uses of circRNAs in CRC

CircRNAs are emerging as critical regulators in oncogenic molecular pathways, interacting with diverse molecules such as miRNAs and lncRNAs (69). Their ability to influence gene expression and cellular behaviors positions them as significant contributors to tumorigenesis and as potential biomarkers for cancer diagnosis and prognosis (54). Specific circRNAs such as circCCDC66 and ciRS-7 have been identified as prognostic biomarkers in CRC. CircCCDC66 is significantly overexpressed in CRC samples and is closely linked to poor patient prognosis (69). Functional analyses reveal that circCCDC66 modulates key oncogenic processes, including cell proliferation, anchorage-independent growth, invasion, and migration, underscoring its pivotal role in CRC progression (69).

A novel diagnostic biomarker for CRC is serum exosomal circRNA, specifically hsa_circ_0004771, which has been detected in CRC patients. Comparative analysis has revealed 39 differentially expressed circRNAs in CRC tissues, with 28 downregulated and 11 upregulated (70). Such distinct expression patterns highlight the potential utility of circRNAs in differentiating CRC from normal tissue. Another circRNA, CircFADS2, derived from the human FADS2 gene, has been strongly correlated with clinicopathological features in CRC. CircFADS2 is highly expressed in over 187 CRC tissue samples, making it a promising candidate for prognostic research (71). Conversely, hsa_circ_0026344 exhibits significantly reduced expression in CRC tissues compared to adjacent non-tumorous tissues (72). It regulates miR-21 and miR-31, suppressing CRC cell growth and adherence while inducing apoptosis. These findings suggest hsa_circ-0026344’s potential as a tumor suppressor and a therapeutic agent in CRC (72).

CircRNAs also influence chemoresistance in CRC through miRNA sponging. For example, ciRS-7 (circular RNA sponge for miR-7) has been associated with advanced T-stage, lymph node involvement, and distant metastases (73). Its overexpression activates the EGFR/RAF1/MAPK pathway by inhibiting miR-7 activity, highlighting its oncogenic role and potential as a therapeutic target (73). Another circRNA, CircPTK2, promotes EMT and metastasis in CRC cells by binding to vimentin, a key EMT regulator. Elevated CircPTK2 levels correlate with shorter survival rates, as Yang et al. (2020) reported, making it a potential target for metastasis control in CRC (74). Additionally, hsa_circ_0001649 has demonstrated oncogenic activity across various cancers, including liver, ovarian, and prostate cancers, underscoring its broader significance in malignancies (75). These circRNAs represent a multifaceted network of regulatory molecules that drive CRC progression and offer opportunities for innovative diagnostic, prognostic, and therapeutic strategies. Their involvement in EMT, drug resistance, and apoptosis regulation processes further strengthens their potential as crucial targets in CRC management (74, 75).

CircRNAs, known for their potential as microRNA sponges and competitive endogenous RNAs, are emerging as important factors in cancer biology (59). Despite their typically unstable expression in tumor cells, circRNAs have shown significant connections to cancer progression, particularly through interactions with miRNAs and proteins. Increased expression of CiRS-7 in CRC cells allows it to function as a microRNA sponge, sequestering miR-7 and upregulating its targets (76). This regulation affects the expression of epidermal growth factor receptors, which are pivotal in controlling cancer cell proliferation, differentiation, and signaling pathways (76). Additionally, a study by Chen et al. (2019) revealed that circNSUN2 with m6A mutation exhibited enhanced cytoplasmic export through YTHDC1 (41). Further findings demonstrated that the circNSUN2/IGF2BP2/HMGA2 RNA-protein ternary complex stabilized HMGA2 mRNA, facilitating CRC metastatic growth. These results highlight potential clinical and therapeutic implications of m6A modifications in circRNA biology (41).

Chang et al. (2023) demonstrated that introducing artificial circRNAs into mammalian cells promoted the expression of innate immunity-related genes, which protected the Venezuelan equine encephalitis virus (77). Similarly, many circRNAs with oncogenic miRNA and protein-binding sites have shown potential in restoring regulated cancer cell growth or inducing apoptosis (78). For example, circRNA0003906 exhibited diagnostic potential in CRC, with a study using an ROC curve to differentiate 122 CRC tissues from 40 healthy controls (79). In exosomal circRNAs, circPACRGL was significantly elevated in CRC cells, promoting proliferation, migration, and invasion. Meanwhile, circSLC7A6 acted as an apoptosis inhibitor and a regulator of CRC cell invasion and growth (80, 81).

Given the challenges of traditional screening methods, such as stool-based assays’ low sensitivity and specificity, high colonoscopy costs, and limited global compliance, circRNAs represent a valuable alternative (82, 83). Numerous circRNAs have shown strong associations with clinicopathological features, underscoring their potential for guiding CRC prognosis and treatment decisions (84). CircRNAs like hsa_circ_0000504 have shown therapeutic promise in clinical practice, targeting miR-885-3p via the AKT signaling pathway (85). Collectively, circRNAs provide a multifaceted platform for advancing CRC diagnostics, prognostics, and therapeutic interventions, offering hope for more effective and accessible patient care worldwide (64, 85).

Some circRNAs can discriminate between early and advanced CRC stages, indicating potential utility for staging cases and offering prognostic value (86). As a less invasive diagnostic tool, circRNAs may provide an alternative approach to CEA and better performance, e.g., CEA-sensitivity plus circRNA-specificity (87). Specific circRNAs have been associated with early and advanced disease, and circRNAs have roles in tumor growth, progression, and metastases (63). CircRNAs play roles in chemotherapy resistance and may modify a patient’s response to treatment decisions and targeted therapies. They also have the potential to be a biomarker of predicting therapy response and monitoring disease progression (54). For example, circ-0084615 and circ-0006174 have influenced tumor growth and metastasis by sequestering microRNAs, while other circRNAs such as CDR1as and circHIPK3 influence CRC progression through sponging tumor-suppressing miRNAs, and promoting oncogenic signaling pathways (53). These research findings demonstrate the roles of circRNAs in CRC as a whole, but also find the potential application of circRNAs in CRC as a diagnostic, prognostic and therapeutic target (53, 54).

Oxaliplatin is a standard chemotherapeutic treatment for colon cancer, but there have been instances of therapeutic resistance, whose potential can be mediated by circRNAs (88). CircRNA hsa_circ_0076691 is overexpressed in oxaliplatin resistant colon cancer cells. It contributes to oxaliplatin resistance by acting as a molecular sponge for miR-589-3p, decreasing its available concentrations while upregulating its downstream target FGF9 (89). For instance, by sequestering miRNAs, circRNAs such as hsa_circ_0076691 adapt miRNA roles in normal regulatory networks controlling cell survival and apoptosis, which is vital for oxaliplatin-induced cytotoxicity. Consequently, circRNAs such as hsa_circ_0076691 can mitigate drug-induced apoptosis, allowing tumor cells to survive and promote chemoresistance (89). This further emphasizes their potential as therapeutic targets; e.g., by inhibiting circRNAs like hsa_circ_0076691, the sensitivity to oxaliplatin can be improved (89).

This is a similar scenario for circRNAs when it comes to 5-FU. 5-FU is an essential chemotherapeutic agent for colon cancer. The development of 5-FU resistance highly reduces its clinical utility, and circRNAs drive 5-FU resistance through direct or indirect mechanisms, particularly through regulating signaling pathways, sponging miRNA, or drug efflux (90). For example, overexpression of circNRIP1 promoted the progression of CRC, while its silencing sensitized CRC cells to 5-FU by sponging miR-532-3p (91). An additional circRNA, circACC1, was linked to increased AMPK activity (92). Increased AMPK activity contributes to increased resistance by promoting pro-survival signaling. Overall, circRNA have a significant involvement of circRNAs in chemotherapy drug resistance (92). **Table 1** demonstrates various circRNAs identified in CRC, their sample types (e.g., blood, plasma, tissue) and their reported sensitivity and specificity as diagnostic biomarkers (93–100).

**Table 1:** Diagnostic performance of circRNAs in CRC.

circRNA	Sample Type	Sensitivity	Specificity	Reference
circ_0013958	Tissue	High	Moderate	(93)
circ_0001445	Plasma	Moderate	Moderate	(94)
circ_0001178	Plasma	High	High	(95)
circ_0000826	Tissue	High	High	(96)
circ_0001313	Tissue	High	Moderate	(97)
circ_0026344	Tissue	High	Moderate	(72)
circ_0035445	Plasma	High	Moderate	(98)
circ_0082182	Tissue	High	High	(99)
circ_0001313	Tissue	High	Moderate	(100)

The ncRNAs, which include circRNAs, miRNAs, and lncRNAs, have become important candidates for diagnostic and prognostic biomarkers for CRC (101). CircRNAs are an exciting subclass of ncRNA that can be considered primarily due to their structure. CircRNAs form discrete covalently closed-loop structures, allowing increased stability and insensitivity to exonuclease digestion. Their half-life can be extremely long in circulation and is abundant compared to other RNA types (102). miRNAs can have distinct advantages for non-invasive cancer diagnostics, even though they are less stable than circRNAs. miRNAs are small, endogenous ncRNAs that regulate gene expression post-transcriptionally, and they are commonly dysregulated in colon cancer (103). Many miRNAs can be studied from body fluids like blood, stool, and urine to promote minimally invasive diagnostic screening and monitoring approaches. miRNAs can be considered tumor suppressors or oncogenes, where their expression levels have been shown to alter throughout stages of CRC development and disease progression (104). The long sequence and complex secondary structures of lncRNAs allows them to bind and interact with DNA, RNA, and proteins, influencing gene expression at the epigenetic, transcriptional, and post-transcriptional levels (105). Dysregulated lncRNAs are involved in tumor proliferation, invasion, metastasis, and treatment resistance in colon cancer. Similarly, both lncRNAs and circRNAs can act as miRNA molecular sponges that can modulate the activities of miRNA-mRNA networks, thereby regulating oncogenic signaling pathways (106).

CircRNAs are stable and more resistant to degradation than linear RNAs, which aids in their use as robust biomarkers; miRNAs are detectable in a non-invasive manner and represent dynamic changes in-state of disease; lncRNAs potentially offer more information than miRNAs due to their considerable range of functional roles in CRC and can give a better understanding of the highly connected regulations of transcription and translation in CRC (107, 108). In addition, the interactions between each class of ncRNAs could add to regulatory pathways and may allow for combinatorial biomarkers (109). Targeting these on developing diagnostic tests that will be sensitive and specific to ncRNAs and CRC may help improve the cancer diagnosis, prediction of disease progression, and treatment regimens based on patients’ specific needs (110).

## 5 Challenges in using circRNAs for CRC diagnosis

Despite the promising potential of circRNAs as diagnostic biomarkers for CRC, several limitations exist in clinical applications. The detection and quantification of circRNAs remains challenging, with many techniques still being developed (111). While next-generation sequencing allows for transcriptome-wide circRNA identification, the reliability of these methods requires meticulous study design and execution (112). Validating circRNAs, particularly demonstrating their circularity and confirming the back-splice junction, presents technical hurdles. The variable abundance of circRNAs, with some highly expressed and others present in low quantities, may affect their consistency as biomarkers (113). Furthermore, the stability and conservation of circRNAs can vary depending on specific conditions, potentially impacting their effectiveness across diverse patient populations. Our understanding of the regulatory mechanisms controlling circRNA synthesis remains limited, thus complicating the efforts to predict or manipulate their expression (113). For instance, the role of environmental factors and microbiota in circRNA expression regulation remains underexplored, posing challenges in developing circRNA-based personalized diagnostics (114).

Integrating circRNA analysis into standard clinical workflows is a significant challenge, as current detection methods are often expensive and technically demanding (115). Ensuring cost-effectiveness and scalability will be critical for the widespread use of circRNA as a biomarker for CRC. Additionally, invasive tissue sampling methods for circRNA detection limit their applicability, underscoring the need for further research into non-invasive approaches, such as liquid biopsies (115, 116). Another critical gap is the lack of robust computational tools for large-scale circRNA analysis. Though promising, AI and machine learning techniques are not yet fully optimized to handle the complexity of circRNA datasets, which may impede their integration into clinical practice (117). Moreover, many circRNAs have been identified, and the functional and clinical significance of most remains unclear, necessitating further research, especially clinical studies, to establish their relevance in CRC diagnosis and therapeutics. Targeted circRNA delivery mechanisms, such as nanoparticle-based systems, are still under development, and their safety and efficacy require validation in preclinical and clinical settings. Addressing these constraints is critical for advancing circRNA-based CRC diagnostics and therapies (118).

## 6 CircRNAs in CRC: future perspectives and concluding insights

Early detection of CRC is crucial for its prevention and significantly impacts long-term patient survival. Numerous dysregulated circRNAs have been identified as potential targets for addressing chemoradiation resistance in CRC treatment. In summary, circRNAs offer potential for the early detection, prognosis, and treatment of CRC. Their utility as non-invasive biomarkers, combined with their involvement in key oncogenic pathways like the AKT signaling pathway, underscores their value in overcoming chemoradiation resistance. CircRNA-based therapeutics, particularly combined with treatments such as 5-FU, hold significant potential. Additionally, advancements in nucleic acid-editing technologies, such as CRISPR, further enhance their therapeutic relevance. Integrating multi-omics approaches and AI-driven models in future research could lead to personalized, more effective diagnostic strategies, ultimately improving long-term outcomes for CRC patients.

Our understanding of circRNAs has been further enhanced by insights from other cancers, revealing conserved oncogenic mechanisms. Cross-cancer studies underscore the potential of circRNAs as universal diagnostic and therapeutic targets, paving the way for multi-cancer diagnostic platforms. Additionally, the influence of environmental and lifestyle factors on circRNA expression opens new avenues for integrating personalized prevention strategies into CRC management. Furthermore, circRNAs have shown promise in predicting treatment response and monitoring therapy potential in CRC. However, despite these findings, significant challenges remain in translating circRNA research into clinical practice, including the need for standardized detection methods, validation in larger patient cohorts, and a deeper understanding of circRNA’s role. Future integration of multi-omics approaches and advanced computational tools, such as AI-based predictive models, could improve circRNA-based diagnostics and enhance personalized CRC treatment.

In conclusion, circRNAs represent a promising frontier in CRC research, offering potential solutions for early detection, personalized treatment, and improved patient outcomes. The synergy between circRNA research and cutting-edge technologies holds the potential of CRC management, ultimately reducing the global burden of this disease. CircRNAs such as ciRS-7, circCCDC66, and hsa_circ_0026416 appear to be effective diagnostic and prognostic factors in CRC given their stability and expression. Their ability to mediate critical pathways and resistance to chemotherapy extensions indicates that circRNAs could be novel targets for CRC. Detection of hsa_circ_0026416 via liquid biopsy will help to develop a non-invasive detection method for early CRC diagnosis and monitoring. Thus circRNAs could serve as a potential way for RNA-based therapeutics and personalized treatment in CRC.

## Glossary

CRC: Colorectal Cancer
CircRNA: Circular RNA
ncRNA: Non-coding RNA
miRNA: MicroRNA
lncRNA: Long Non-coding RNA
ceRNA: Competing Endogenous RNA
RBP: RNA-Binding Protein
RT-qPCR: Reverse Transcription Quantitative Polymerase Chain Reaction
RNA-seq: RNA Sequencing
EMT: Epithelial-Mesenchymal Transition
FIT: Fecal Immunochemical Test
g-FOBT: Guaiac Fecal Occult Blood Test
CEA: Carcinoembryonic Antigen
EGFR: Epidermal Growth Factor Receptor
CDR1as: Cerebellar Degeneration-Related Protein 1 Antisense
PET/CT: Positron Emission Tomography/Computed Tomography
CT: Computed Tomography
ROC: Receiver Operating Characteristic
AUC: Area Under the Curve
EV: Extracellular Vesicle
BSS: Back-Splice Site
EIB: Exon-Intron Boundary
UTR: Untranslated Region
m6A: N6-Methyladenosine
HMGA2: High Mobility Group AT-Hook 2
IGF2BP2: Insulin-like Growth Factor 2 mRNA Binding Protein 2
YTHDC1: YTH Domain Containing 1
HNPCC: Hereditary Non-Polyposis Colorectal Cancer
FAP: Familial Adenomatous Polyposis
IBD: Inflammatory Bowel Disease
5-FU: 5-Fluorouracil
AMPK: AMP-Activated Protein Kinase
snRNA: Small Nuclear RNA
CTIC1: Colorectal Tumor Initiating Cell-1 (circCTIC1)
AKT: Protein Kinase B (PKB/AKT pathway)
MAPK: Mitogen-Activated Protein Kinase
RAF1: Proto-oncogene serine/threonine-protein kinase
CRISPR: Clustered Regularly Interspaced Short Palindromic Repeats
DNA: Deoxyribonucleic Acid
RNA: Ribonucleic Acid
snRNP: Small Nuclear Ribonucleoprotein
UTRs: Untranslated Regions
OS: Overall Survival
CI: Confidence Interval
NCT: National Clinical Trial.
